# Intracellular iron deficiency in pulmonary arterial smooth muscle cells induces pulmonary arterial hypertension in mice

**DOI:** 10.1073/pnas.1822010116

**Published:** 2019-05-31

**Authors:** Samira Lakhal-Littleton, Alexi Crosby, Matthew C. Frise, Goran Mohammad, Carolyn A. Carr, Paul A. M. Loick, Peter A. Robbins

**Affiliations:** ^a^Department of Physiology, Anatomy and Genetics, University of Oxford, OX1 3PT Oxford, United Kingdom;; ^b^Department of Medicine, University of Cambridge Addenbrooke’s Hospital, CB2 0QQ Cambridge, United Kingdom

**Keywords:** iron, ferroportin, hepcidin, pulmonary arterial hypertension, endothelin-1

## Abstract

Iron deficiency augments hypoxic pulmonary arterial pressure in healthy individuals and exacerbates pulmonary arterial hypertension (PAH) in patients, even without anemia. Conversely, iron supplementation has been shown to be beneficial in both settings. The mechanisms underlying the effects of iron availability are not known, due to lack of understanding of how cells of the pulmonary vasculature respond to changes in iron levels. The iron export protein ferroportin (FPN) and its antagonist peptide hepcidin control systemic iron levels by regulating release from the gut and spleen, the sites of absorption and recycling, respectively. We found FPN to be present in pulmonary arterial smooth muscle cells (PASMCs) and regulated by hepcidin cell autonomously. To interrogate the importance of this regulation, we generated mice with smooth muscle-specific knock in of the hepcidin-resistant isoform fpn C326Y. While retaining normal systemic iron levels, this model developed PAH and right heart failure as a consequence of intracellular iron deficiency and increased expression of the vasoconstrictor endothelin-1 (ET-1) within PASMCs. PAH was prevented and reversed by i.v. iron and by the ET receptor antagonist BQ-123. The regulation of ET-1 by iron was also demonstrated in healthy humans exposed to hypoxia and in PASMCs from PAH patients with mutations in bone morphogenetic protein receptor type II. Such mutations were further associated with dysregulation of the HAMP/FPN axis in PASMCs. This study presents evidence that intracellular iron deficiency specifically within PASMCs alters pulmonary vascular function. It offers a mechanistic underpinning for the known effects of iron availability in humans.

## Significance

Pulmonary arterial hypertension (PAH) is a disease in which lung blood pressure is raised chronically, causing right heart failure. It has been shown that iron deficiency also raises lung blood pressure. However, we don’t know the mechanisms because we don’t understand precisely how cells of the lung blood vessels are affected by iron levels. The smooth muscle cells of the lung blood vessels are important for controlling lung blood pressure. Our study shows that iron deficiency specifically within these cells is sufficient to cause PAH, even against a background of normal iron levels in other tissues.

Iron deficiency is the most common nutritional disorder in the world (1). It is highly prevalent in patients with pulmonary arterial hypertension (PAH), where it is associated with greater disease severity (2–5). At the same time, iron supplementation has been shown to attenuate the normal pulmonary vasoconstrictive response to hypoxia and to improve some functional measures in PAH patients regardless of the presence or absence of anemia (6–12). The mechanisms underlying the effects of iron deficiency and iron supplementation on pulmonary vascular function remain unclear, due to a gaping hole in our understanding of how cells of the pulmonary vasculature respond to changes in iron levels (13).

The healthy functioning of tissues requires tight control of intracellular iron levels. These in turn are dependent both on cellular homeostatic pathways controlling iron uptake, usage, storage, and release, and on systemic pathways controlling iron availability in the plasma. In mammals, cellular iron homeostasis is controlled by the iron regulatory proteins (IRP), which controls the levels of iron uptake proteins such as transferrin receptor 1 (TfR1) and divalent metal transporter 1 (DMT1), the iron storage protein ferritin, and in some cells the iron export protein ferroportin (FPN) (14, 15). Systemic iron homeostasis is controlled by the hepcidin/FPN axis at the sites of iron entry into the circulation. FPN is the only known mammalian iron export protein and mediates iron release into the circulation from the gut, spleen, and liver, the sites of iron absorption, recycling, and storage, respectively (16–18). FPN is antagonized by the hormone hepcidin, also known as hepcidin antimicrobial peptide (HAMP). Produced primarily in the liver, HAMP binds to and induces internalization of FPN, thereby limiting iron release into the circulation and its availability to peripheral tissues (16–18). The importance of the systemic HAMP/FPN axis is illustrated by diseases of systemic iron overload such as hemochromatosis and β-thalassaemia, where HAMP production or responsiveness is impaired (19, 20) and in the anemia of chronic inflammation where HAMP production is inappropriately elevated (21). FPN and HAMP are also found in cells with no recognized role in systemic iron homeostasis, including pulmonary arterial smooth muscle cells (PASMCs) and cardiomyocytes (22–24).

Additionally, data acquired during a previous study of a mouse model of the iron overload disease hemochromatosis, generated through a ubiquitous knock in (KI) of the fpnC326Y gene (which encodes a hepcidin-resistant FPN), revealed that FPN was markedly up-regulated in PASMCs compared with lungs from wild-type control mice (*SI Appendix*, Fig. S1*A*). In that setting, it could not be discerned if FPN up-regulation was due to loss of HAMP responsiveness in PASMCs or to an IRP-driven response to intracellular iron overload. To eliminate the confounding effects of iron overload, we generated a mouse model with an inducible smooth muscle cell-specific knock in of fpnC326Y and found that FPN was still up-regulated in PASMCs from these mice, confirming that the HAMP/FPN operates in PASMCs. We then set out to establish the physiological importance of the HAMP/FPN axis in PASMCs. To this end, we studied pulmonary vascular hemodynamics and cardiac function longitudinally in these mice.

## Results

### Loss of HAMP Responsiveness in PASMCs Leads to PAH and Right Heart Failure.

We generated mice with an inducible smooth muscle cell-specific KI of the HAMP-resistant isoform fpnC326Y as described in *SI Appendix*, using mice transgenic for Y chromosome-linked CreER^T2^ gene under the control of the smooth muscle myosin heavy chain polypeptide 11 (myh11) promoter (25). Male fpnC326Y^fl/fl^ SMMHC-CreER^T2+^ and fpnC326Y^fl/fl^ controls were induced with tamoxifen in the first week after weaning. Consistent with the smooth muscle-specific nature of the myh11-driven cre recombinase, and with the notion that FPN in smooth muscle cells is not involved in systemic iron homeostasis, fpnC326Y^fl/fl^ SMMHC-CreER^T2+^ mice did not differ from age-matched fpnC326Y^fl/fl^ controls in terms of serum and tissue iron indices, unlike ubiquitous fpnC326Y-KI mice which show the classical hemochromatosis phenotype (*SI Appendix*, Fig. S1 *B*–*D*).

**Fig. 1.**
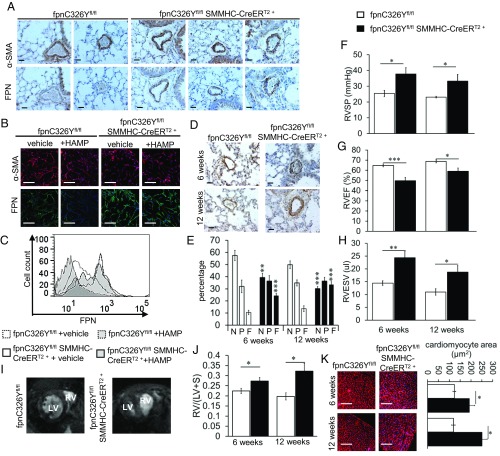
Loss of HAMP responsiveness in PASMCs leads to PAH and right heart failure. (*A*) Representative images of α-SMA and FPN immunostaining in the lung 6 wk posttamoxifen induction (*n* = 6 mice per group). Original magnification 40×. (Scale bar: 25 μm.) (*B*) Representative images of α-SMA (red) and FPN (green) immunostaining in primary mouse PASMCs. Cells were treated with either vehicle or exogenous HAMP peptide (+HAMP). Original magnification 20×. (Scale bar: 100 μm.) (*n* = 3 mice) (*C*) Membrane FPN levels measured by flow cytometry in primary mouse PASMCs (*n* = 3 mice). (*D*) Representative images of α-SMA immunostaining in the lungs at 6 and 12 wk posttamoxifen induction (*n* = 6 per group). Original magnification 40×. (Scale bar 25: μm.) (*E*) Percentage of nonmuscularized (N), partially muscularized (P), and fully muscularized (F) vessels in lungs at 6 wk and 12 wk posttamoxifen induction (*n* = 5–6 per group). *P* values shown relative to the respective vessel category in control mice of the respective time point. (*F*–*H*) Measurements of right ventricular systolic pressure (RVSP) (*n* = 5–6), right ventricular ejection fraction (RVEF) (*n* = 5–12) and right ventricular end systolic volume (RVESV) (*n* = 5–12). (*I*) Representative midventricular magnetic resonance images of the heart showing septal bowing at 6 wk post tamoxifen induction (*n* = 5–12 per group). (*J*) Ratio of RV to LV+Septum weight (*n* = 5–6 per group). (*K*) Representative images of WGA staining in RV (*n* = 5–6 per group). Original magnification 10×. (Scale bar: 200 μm.) Quantitation of cardiomyocyte area shown in *Right*. *P* values for paired comparisons were calculated using Student’s *t* test. *P < 0.05, **P < 0.01, ***P < 0.001.

We then characterized FPN levels in PASMCs of fpnC326Y^fl/fl^ SMMHC-CreER^T2+^ mice. Immunostaining for FPN in the lungs showed marked FPN up-regulation in PASMCs of fpnC326Y^fl/fl^ SMMHC-CreER^T2+^ animals compared with control fpnC326Y^fl/fl^ mice (Fig. 1*A*). The increase in FPN levels appeared to be confined to the pulmonary vasculature, as no change was detected in the smooth muscle cells of the aorta or heart (*SI Appendix*, Fig. S2*A*).

When we isolated primary PASMCs, we found that FPN was more highly expressed in cells from fpnC326Y^fl/fl^ SMMHC-CreER^T2+^ mice than in cells from fpnC326Y^fl/fl^ controls. Furthermore, in vitro treatment with exogenous HAMP peptide resulted in down-regulation of FPN protein in fpnC326Y^fl/fl^ PASMCs but not in fpnC326Y^fl/fl^ SMMHC-CreER^T2+^ PASMCs, consistent with the HAMP-resistant nature of the C326Y mutation (Fig. 1 *B* and *C*). Together, these data confirm that FPN in PASMCs is directly regulated by HAMP.

Having established that FPN in PASMCs is regulated by HAMP, we then assessed the physiological importance of such regulation. First, we examined the pulmonary vasculature in fpnC326Y^fl/fl^ SMMHC-CreER^T2+^ and fpnC326Y^fl/fl^ males at two time points posttamoxifen injection, 6 and 12 wk. Immunostaining for the smooth muscle marker, smooth muscle cell actin-alpha (α-SMA) revealed some thickening of the smooth muscle layer and increased muscularization of pulmonary arteries in fpnC326Y^fl/fl^ SMMHC-CreER^T2+^ mice (Fig. 1 *D* and *E*). To explore pulmonary hemodynamics, we measured right ventricular systolic pressure (RVSP) in anesthetized mice. We found that fpnC326Y^fl/fl^ SMMHC-CreER^T2+^ mice had elevated RVSP compared with fpnC326Y^fl/fl^ controls at both time points (Fig. 1*F*).

Changes in the pulmonary vasculature are known to influence the function of the right ventricle. Therefore, we measured parameters of cardiac function in live anesthetized mice by cine MRI. We found that, compared with fpnC326Y^fl/fl^ controls, fpnC326Y^fl/fl^ SMMHC-CreER^T2+^ mice had reduced RV ejection fraction (RVEF) and increased RV end systolic volume (RVESV) at both time points (Fig. 1 *G* and *H*). Parameters of LV function were not different between mice of the two genotypes (*SI Appendix*, Table S1). Cine MR imaging also showed that 5 of the fpnC326Y^fl/fl^ SMMHC-CreER^T2+^ mice (*n* = 12), but none of the fpnC326Y^fl/fl^ controls (*n* = 5), developed visible septal bowing (Fig. 1*I*). These changes in RV function were associated with RV hypertrophy as determined by quantitation of RV weight and cardiomyocyte size (Fig. 1 *J* and *K*). Together, these results demonstrate that fpnC326Y^fl/fl^ SMMHC-CreER^T2+^ mice develop PAH and right ventricular remodelling.

### PAH in fpnC326Y^fl/fl^ SMMHC-CreER^T2+^ Mice Is Caused by Intracellular Iron Deficiency in PASMCs and Is Prevented and Reversed by i.v. Iron Treatment.

**Fig. 2.**
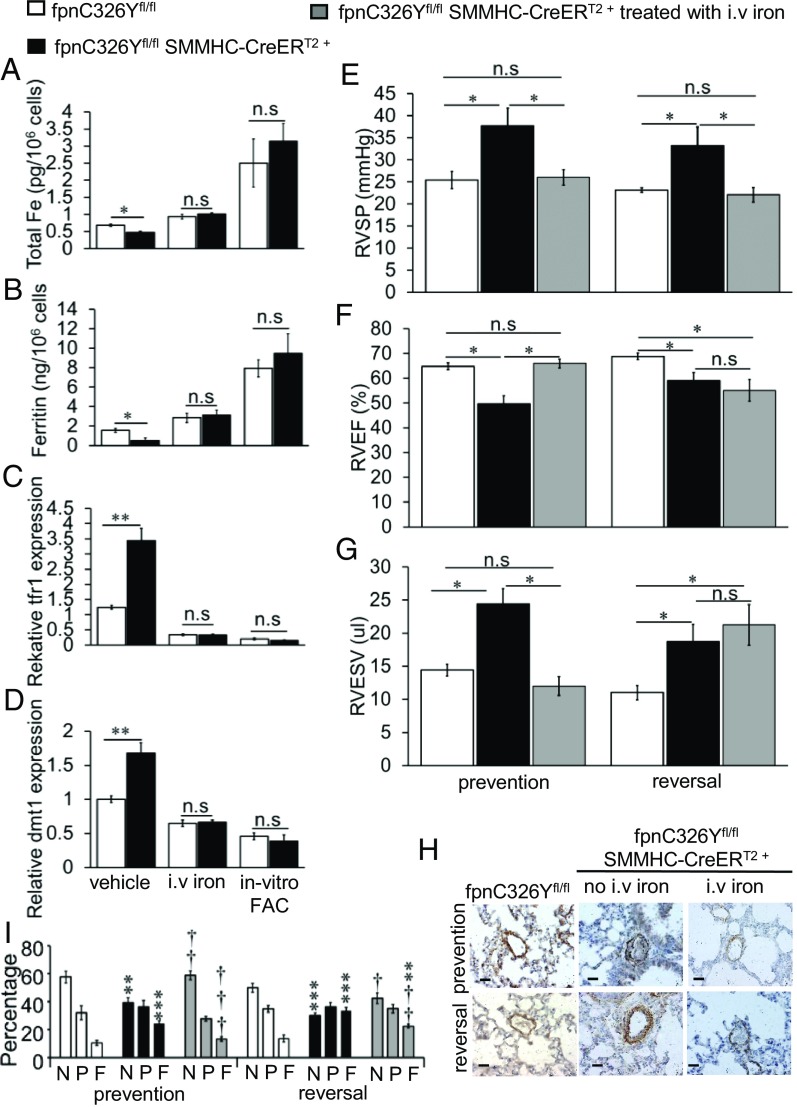
PAH in fpnC326Y^fl/fl^ SMMHC-CreER^T2+^ mice is caused by intracellular iron deficiency in PASMCs and is prevented and reversed by i.v. iron treatment. (*A*–*D*) Total elemental iron, cellular ferritin, relative tfr1expression and relative dmt1 expression in primary PASMCs 6 wk posttamoxifen induction. Cells were either isolated from non-iron–treated mice, then cultured with vehicle or in the presence ferric citrate (in-vitro FAC), or from mice treated with i.v. iron. *n* = 3 mice per group, *n* = 3 biological replicates per mouse. (*E*–*G*) Measurements of RVSP, RVEF and RVESV in iron-treated fpnC326Y^fl/fl^ SMMHC-CreER^T2+^ mice in prevention group (*n* = 5) and reversal group (*n* = 5). Data for non-iron–treated mice at the respective time point are also shown (as per Fig. 1 *F*–*H*). (*H*) Representative images of α-SMA immunostaining in the lungs (*n* = 5–6 per group). Staining in lungs from non-iron–treated mice at the respective time point are also shown (as per Fig. 1*D*). Original magnification 40×. (Scale bar: 25 μm.) (*I*) Percentage of nonmuscularized (N), partially muscularized (P) and fully muscularized (F) vessels (*n* = 5–6 per group). Data from non-iron–treated mice at the respective time point are also shown (as per Fig. 1*E*). ^†^*P* values are relative to nontreated fpnC326Y^fl/fl^ SMMHC-CreER^T2+^ mice at the respective time point. *P values are relative to nontreated fpnC326Y^fl/fl^ mice at the respective time point. (*A*–*D*) *P* values for paired comparisons were calculated using Student’s *t* test. (*E*–*G* and *I*) *P* values were calculated using ANOVA. Post hoc tests used Bonferroni correction to allow for multiple comparisons. ^†^*P* < 0.05, ^††^*P* < 0.01, ^†††^*P* < 0.001, *P < 0.05, **P < 0.01, ***P < 0.001.

To understand the role of iron in the development of PAH in these mice, we compared the iron status of PASMCs from fpnC326Y^fl/fl^ SMMHC-CreER^T2+^ mice and PASMCs from fpnC326Y^fl/fl^ controls, by measuring levels of elemental cellular iron, cellular ferritin, and expression of IRP-regulated genes. We found that both cellular iron and ferritin levels were reduced in PASMCs isolated from fpnC326Y^fl/fl^ SMMHC-CreER^T2+^ mice (Fig. 2 *A* and *B*). Consistent with this, tfr1 and dmt1 were also up-regulated (Fig. 2 *C* and *D*). The reduction in cellular iron and ferritin levels and the up-regulation of tfr1 and dmt1 were not seen in cells derived from mice that had received three fortnightly i.v. injections of 0.5 mg iron (i.v. iron), and in cells treated in vitro with ferric citrate (in vitro FAC). These data demonstrate that PASMCs from fpnC326Y^fl/fl^ SMMHC-CreER^T2+^ mice are relatively iron deficient, and that this intracellular iron deficiency can be corrected in vivo by i.v. iron treatment.

Having established that i.v. iron treatment in vivo can correct intracellular iron levels in PASMCs, we tested whether it could also prevent or reverse PAH in fpnC326Y^fl/fl^ SMMHC-CreER^T2+^ mice. To test this hypothesis, fpnC326Y^fl/fl^ SMMHC-CreER^T2+^ mice were given a total of three fortnightly i.v. injections, each delivering 0.5 mg iron in the form of ferric carboxymaltose. Iron treatment was started either 1 d before the first tamoxifen injection (prevention group) or 6 wk posttamoxifen injection (reversal group). Cardiac function, RVSP, and lung histology were then assessed 6 wk later.

We found that in both groups, iron-treated fpnC326Y^fl/fl^ SMMHC-CreER^T2+^ mice had lower RVSP than non-iron–treated fpnC326Y^fl/fl^ SMMHC-CreER^T2+^ mice of the respective time point (Fig. 2*E*). Additionally, the thickening of the smooth muscle layer and muscularization of the pulmonary arteries were prevented and at least partially reversed by iron treatment (Fig. 2 *H* and *I*). With respect to right heart function, iron treatment prevented but did not reverse the remodelling of the right ventricle (decreased RVEF, increased RVESV) seen in non-iron–treated fpnC326Y^fl/fl^ SMMHC-CreER^T2+^ mice (Fig. 2 *F* and *G*).

Together, these data demonstrate that intracellular iron deficiency in PASMCs from fpnC326Y^fl/fl^ SMMHC-CreER^T2+^ mice causes PAH, and that replenishment of intracellular iron levels through i.v. iron treatment prevents and at least partially reverses the development of PAH. Right heart remodelling in fpnC326Y^fl/fl^ SMMHC-CreER^T2+^ mice appears to be preventable but not reversible by i.v. iron treatment.

### Intracellular Iron Deficiency in PASMCs Causes PAH by Increasing Endothelin-1 Levels.

Having established that iron deficiency in PASMCs caused PAH, we then explored the underlying mechanisms. In a previous study of systemic iron deficiency, we had observed that endothelin-1 (ET-1) was markedly up-regulated in the lungs, including in the pulmonary arteries of mice fed an iron-deficient diet, and that this was prevented by administration of i.v. iron (*SI Appendix*, Fig. S3*A*). Similar changes were seen in vitro following treatment of mouse and human PASMCs with the iron chelator desferroxamine (DFO) and ferric citrate (FAC) (*SI Appendix*, Fig. S3*B*). This observation, together with the known function of ET-1 as a potent endogenous vasoconstrictor led us to hypothesize that ET-1 may contribute to the development of PAH in fpnC326Y^fl/fl^ SMMHC-CreER^T2+^ mice.

**Fig. 3.**
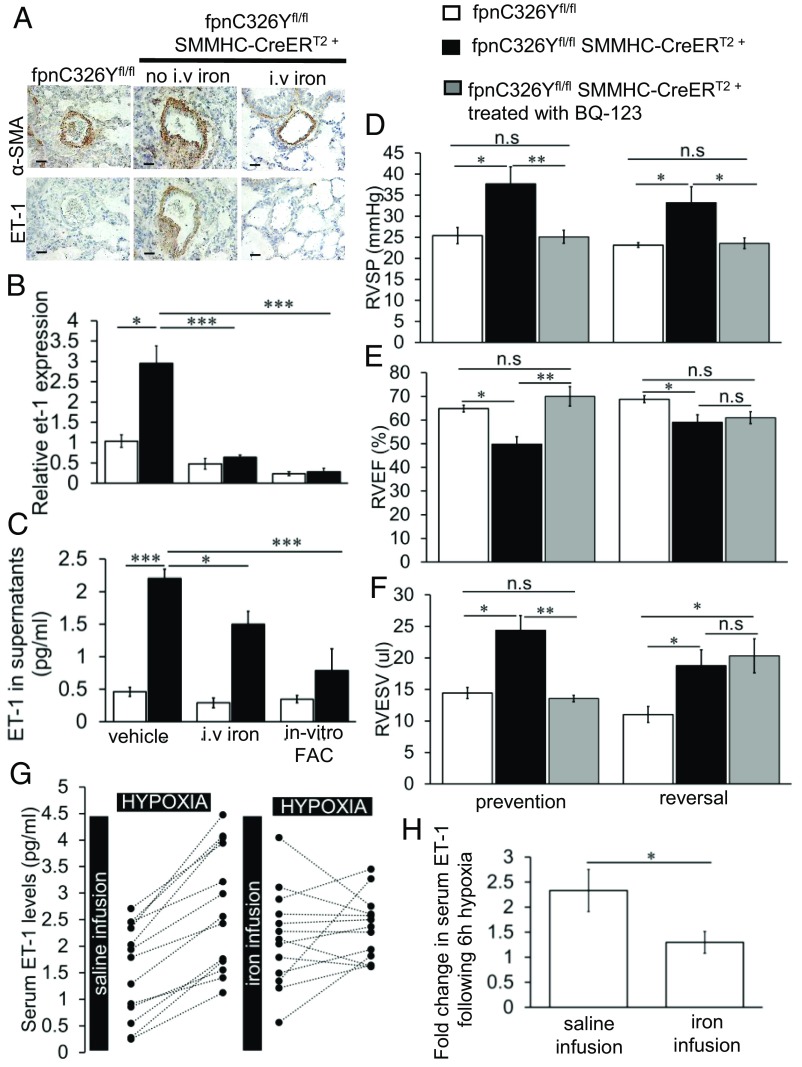
Intracellular iron deficiency in PASMCs causes PAH by increasing ET-1 levels. (*A*) Representative images of α-SMA and ET-1 immunostaining in the lungs of iron-treated fpnC326Y^fl/fl^ SMMHC-CreER^T2+^ mice. Staining in lungs of non-iron–treated mice at the respective time point are also shown. Original magnification 40×. (Scale bar: 25 μm.) *n* = 5–6 per group. (*B*–*C*) Relative expression of et-1 mRNA, and levels of ET-1 protein in supernatants of primary PASMCs isolated from fpnC326Y^fl/fl^ SMMHC-CreER^T2+^ mice and fpnC326Y^fl/fl^ controls 6 wk posttamoxifen induction. Cells were either isolated from non-iron–treated mice, cultured with vehicle, or in the presence of ferric citrate (in vitro FAC), or from mice treated with i.v. iron. *n* = 3 mice per group, *n* = three biological replicates per mouse. (*D*–*F*) Measurements of RVSP, RVEF, and RVESV in BQ-123-treated fpnC326Y^fl/fl^ SMMHC-CreER^T2+^ mice in prevention group (*n* = 6) and reversal group (*n* = 5). Data from non-BQ-123-treated mice at the respective time point are also shown (as per Fig. 1 *F*–*H*).(*G*) ET-1 levels in the sera of 13 healthy volunteers at the start and end of 6 h of eucapnic hypoxia. Volunteers received a saline infusion just before the first exposure and an i.v. iron infusion delivering 15 mg/kg ferric carboxymaltose just before the second exposure. (*H*) Fold change in serum ET-1 levels following hypoxia exposure. (*B*–*F*) *P* values were calculated using ANOVA. Post hoc tests used Bonferroni correction to allow for multiple comparisons. (*H*) *P* values for paired comparisons were calculated using Student’s *t* test. *P < 0.05, **P < 0.01, ***P < 0.001.

To test this hypothesis, we first examined ET-1 expression in the pulmonary arteries. We found that ET-1 was markedly increased in the smooth muscle layer of pulmonary arteries in fpnC326Y^fl/fl^ SMMHC-CreER^T2+^ mice (Fig. 3*A*). Furthermore, iron treatment decreased ET-1 expression in fpnC326Y^fl/fl^ SMMHC-CreER^T2+^ mice (Fig. 3*A*). ET-1 expression in smooth muscle cells of the aorta and heart was not different between mice of different genotypes (*SI Appendix*, Fig. S2*A*).

In accordance with this, when we compared primary PASMCs isolated from fpnC326Y^fl/fl^ SMMHC-CreER^T2+^ mice with those isolated from fpnC326Y^fl/fl^ controls, we found that they expressed higher levels of et-1 mRNA (Fig. 3*B*) and ET-1 protein in supernatants (Fig. 3*C*). Both et-1 mRNA and ET-1 protein in supernatants were lower in cells derived from mice that had received i.v. iron and in cells treated in vitro with ferric citrate (in vitro FAC).

To establish whether this increase in ET-1 levels promoted the development of PAH, we treated fpnC326Y^fl/fl^ SMMHC-CreER^T2+^ mice with the cyclic peptide BQ-123, a selective endothelin subtype-A receptor antagonist using slow release osmotic minipumps. The first group of mice was implanted with the minipumps 72 h before the first tamoxifen injection and assessed at the 6-wk time point (prevention group), while the second group was implanted 8 wk post the first tamoxifen injection and assessed at the 12-wk time point (reversal group).

We found that BQ-123 prevented and reversed the elevation in RVSP seen in non-BQ-123-treated fpnC326Y^fl/fl^ SMMHC-CreER^T2+^ mice of the respective time point (Fig. 3*D*). As with the i.v. iron treatment, the remodelling of the right ventricle was prevented but not reversed by BQ-123 treatment (Fig. 3 *E* and *F*). These findings demonstrate that PASMC-derived ET-1 drives the development of PAH in fpnC326Y^fl/fl^ SMMHC-CreER^T2+^ mice, and that antagonism of the ET_A_ receptor can both prevent and reverse the development of PAH.

Having established a link between iron status and ET-1 in the context of PAH, we then examined the same link in the context of normal pulmonary vascular function. Therefore, we tested ET-1 levels in plasma collected in a previous study of healthy volunteers exposed to 6 h of eucapnic hypoxia on two separate occasions. The first exposure was preceded by a saline infusion, while the second exposure was preceded by i.v. iron infusion of ferric carboxymaltose (7). We found that i.v. iron infusion in the second exposure prevented the hypoxia-driven increase in ET-1 levels that was seen in the first exposure (Fig. 3 *G* and *H*). These results confirm that ET-1 levels are suppressed acutely by i.v. iron treatment and further suggest that such suppression may account for the blunting of the normal pulmonary vascular response to hypoxia.

### The HAMP/FPN Axis Operates Cell Autonomously in PASMCs and Is Dysregulated by BMPR2 Mutations.

**Fig. 4.**
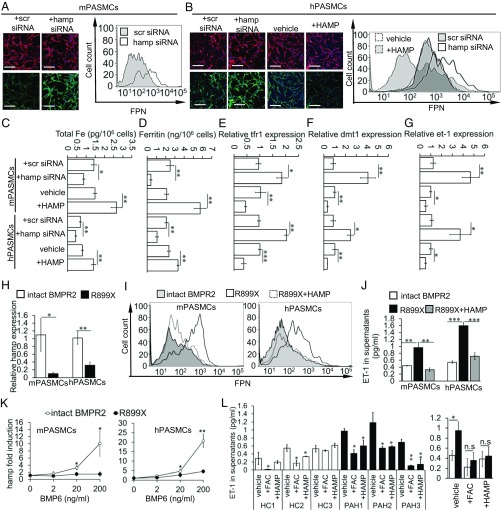
The HAMP/FPN axis operates cell autonomously in PASMCs and is dysregulated by BMPR2 mutations. (*A*) Representative images of FPN (green) and α-SMA (red) staining (*Left*) and FPN flow cytometry (*Right*) in mouse PASMCs. Original magnification 20×. (Scale bar: 100 um.) (*B*) Representative images of FPN (green) and α-SMA (red) staining (*Left*) and FPN flow cytometry (*Right*) in human PASMCs. Cells treated with vehicle or exogenous HAMP peptide (+HAMP) are also shown. Original magnification 20×. (Scale bar: 100 um.) (*C*–*G*) Levels of total elemental iron, cellular ferritin, and relative levels of tfr1, dmt1, and et-1 in mouse and human PASMCs treated with scrambled siRNA, hamp siRNA, vehicle, or HAMP peptide (+HAMP). (*H*) Relative levels of hamp mRNA expression in mouse and human PASMCs with intact BMPR2 or with the R899X mutation. (*I*) Representative images of FPN flow cytometry in mouse and human PASMCs. R899X cells were treated with vehicle (R899X) or exogenous HAMP peptide (R899X+HAMP). (*J*) ET-1 levels in supernatants from corresponding samples. (*K*) Fold induction by BMP6 of hamp mRNA in human and mouse PASMCs with intact BMPR2 or with the R899X mutation. *P* values are shown relative to R899X cells for the respective BMP6 concentration. (*L*) ET-1 levels in the supernatants of primary PASMCs derived from three healthy controls (HC1, -2, and -3) and three PAH patients with known BMPR2 mutations (PAH1, -2, and -3). Cells were treated with either vehicle, FAC, or HAMP peptide. *P* values shown are relative to vehicle-treated cells. (*Right*) Mean values for all HC vs. all PAH. (*E*–*J* and *K*) *P* values for paired comparisons were calculated using Student’s *t* test. (*J* and *L*) *P* values were calculated using ANOVA. Post hoc tests used Bonferroni correction to allow for multiple comparisons. *P < 0.05, **P < 0.01, ***P < 0.001. *n* = 3 biological replicates.

Previously, we showed that exogenous HAMP peptide reduced FPN levels in mouse PASMCs (Fig. 1*B*). Next, we examined the levels and function of endogenous HAMP. We confirmed that HAMP protein is present within the cytoplasm of mouse and human PASMCs (*SI Appendix*, Fig. S4*A*) and released into the supernatants (*SI Appendix*, Fig. S4*B*). Both intracellular HAMP and supernatant HAMP levels were decreased by hamp siRNA treatment compared with scrambled siRNA treatment (*SI Appendix*, Fig. S4 *A* and *B*). We also found that FPN levels in PASMCs were increased by hamp siRNA treatment in mouse PASMCs (Fig. 4*A*) and human PASMCs (Fig. 4*B*). Additionally, as previously observed with mouse PASMCs, exogenous HAMP peptide reduced FPN levels in human PASMCs (Fig. 4*B*). Cellular iron and ferritin content were decreased by hamp siRNA and increased by exogenous HAMP peptide (Fig. 4 *C* and *D*). Expression of tfr1 and dmt1 were increased by Hamp siRNA and decreased by exogenous HAMP peptide, consistent with an IRP-mediated response to changes in intracellular iron levels (Fig. 4 *E* and *F*). Additionally, et-1 expression was increased by hamp siRNA and decreased by exogenous HAMP peptide (Fig. 4*G*). Together, these data demonstrate that HAMP in PASMCs regulates FPN levels, intracellular iron content, and et-1 expression in a cell-autonomous manner.

In the context of the liver, BMPR2 is an important regulator of hepcidin gene expression (26). At the same time, some BMPR2 mutations are associated with the development of familial PAH (27). Therefore, we examined the effect of one such mutation (R899X) on hamp mRNA levels in human and mouse PASMCs. We found that PASMCs with the R899X mutation expressed lower basal levels of hamp mRNA than PASMCs with intact BMPR2 (Fig. 4*H*). Consistent with this, the R899X mutation was associated with higher levels of FPN expression on PASMCs (Fig. 4*I*) and ET-1 in their supernatants (Fig. 4*J*). Treatment of R899X PASMCs with exogenous HAMP peptide reduced both FPN and ET-1 levels (Fig. 4 *I* and *J*). In the liver, BMP6 is the main agonist for hamp mRNA induction through BMPR2. Therefore, we examined the effect of the R899X mutation on hamp induction by BMP6. We found that BMP6 dose-dependently increased hamp mRNA in PASMCs with intact BMPR2, but failed to affect hamp mRNA levels in PASMCs with the R899X mutation (Fig. 4*K*). Taken together, these data indicate that the cellular HAMP/FPN axis in PASMCs is dysregulated by BMPR2 mutations, leading to intracellular iron deficiency, and elevated ET-1 levels. Increased ET-1 levels have been implicated in the etiology of PAH (28, 29). Consistent with this, we found that ET-1 levels were higher in the supernatants of PASMCs from PAH patients with known BMPR2 mutations, than in the supernatants of PASMCs from healthy controls, and that these were reduced following supplementation of the growth medium with iron (+FAC) or exogenous HAMP peptide (Fig. 4*L*).

## Discussion

**Fig. 5.**
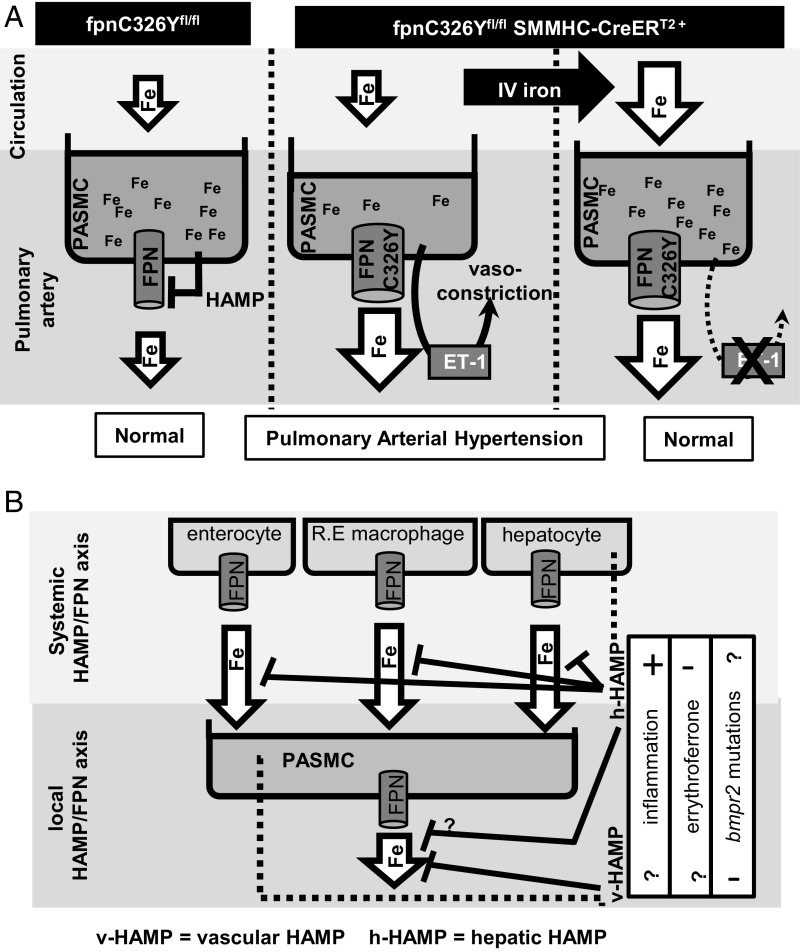
Iron homeostasis and the pulmonary vasculature. (*A*) Mechanism of PAH development in fpnC326Y^fl/fl^ SMMHC-CreER^T2+^ mice. Intracellular iron levels in PASMCs are a balance between iron release (controlled cell autonomously by the HAMP/FPN axis) and iron uptake (dependent on circulating iron levels). Expression of FPNC326Y tips this balance toward iron release, intracellular iron depletion, and up-regulation of ET-1 in PASMCs ET-1. Iron treatment by i.v. redresses the balance by increasing circulating iron availability, thereby replenishing intracellular iron levels and decreasing ET-1 release from PASMCs. (*B*) Schematic model of the interplay between the systemic and the local HAMP/FPN axes in controlling intracellular iron levels in PASMCs.

The major finding of the current study is that intracellular iron deficiency specifically in PASMCs, caused by dysregulation of their FPN, leads to PAH and right heart failure. PAH and right heart failure occurred against a background of otherwise intact systemic iron homeostasis and in the absence of anemia. This study is a demonstration of a causal relationship between intracellular iron deficiency and PAH (Fig. 5*A*).

The effects of systemic iron deficiency on the pulmonary vasculature have been demonstrated both in the context of normal physiological responses and in the setting of PAH. It has been shown that clinical iron deficiency as well as acute infusion with the iron chelator DFO enhance the pulmonary vasoconstrictive response to hypoxia (6–9). Iron deficiency is also prevalent in PAH, and iron-deficient PAH patients have been found to have a reduced performance in the 6-min walk test (6MWT) compared with iron-replete PAH patients (12). Other studies found that iron deficiency in PAH patients was associated with increased disease severity and poor clinical outcome (2, 5). Additionally, iron deficiency is associated with elevated PAP in other disease settings (30, 31). The precise mechanisms underlying the effects of systemic iron deficiency on the pulmonary vasculature have remained unclear, owing to the lack of experimental models that can discern the contribution of anemia (acting systemically through reduced tissue oxygenation and exercise capacity) from that of local iron deficiency acting directly in the vascular tissue. A study using dietary iron restriction in rats, found that such rats developed PAH associated with increased levels of hypoxia-inducible factors (HIFs) in the lung within 2 wk (32). In that setting, rats also developed anemia and reduced hematocrit in the first week. The advantage afforded by the use of fpnC326Y^fl/fl^ SMMHC-CreER^T2+^ mice, is that it allows probing for the effect of local iron deficiency in the vascular tissue without the confounding effects of anemia. We also explored the possibility that local iron deficiency in the vascular tissue and anemia act cooperatively to alter pulmonary vascular function. However, we found that dietary iron restriction, with concomitant anemia did not produce a further rise in RVSP in fpnC326Y^fl/fl^ SMMHC-CreER^T2+^ mice, demonstrating that PASMC iron deficiency fully explains the effects of systemic iron deficiency on the pulmonary vasculature (*SI Appendix*, Fig. S5).

The second important finding of this study is that the potent endogenous vasoconstrictor ET-1 is regulated by iron. The roles of ET-1 in normal vascular responses to hypoxia and in the etiology of PAH have long been recognized. ET-1 is elevated in the lung and in the circulation of PAH patients (28, 29), and a number of trials have demonstrated the benefits of ET receptor antagonists in this disease (33). The present findings that ET-1 is up-regulated in PASMCs from FpnC326Yfl/fl SMMHC-CreER^T2+^ mice, in the serum of healthy volunteers following acute hypoxia exposure, and in the supernatants of PASMCs from PAH patients are all consistent with this long-recognized role. Importantly, the findings that i.v. iron treatment reduced ET-1 in the lungs of fpnC326Y^fl/fl^ SMMHC-CreER^T2+^ mice, and in the serum of acutely hypoxic individuals, and that in vitro iron treatment decreased ET-1 in PASMCs from PAH patients, provide a mechanism to underpin the benefits of iron supplementation reported in human studies. Indeed, iron infusion has been shown to decrease the magnitude of the normal acute hypoxic response in healthy individuals, attenuate the exaggerated hypoxic response in iron-deficient individuals, and improve the performance in the 6MWT in PAH patients (6–12). That i.v. iron acts directly on the pulmonary vasculature to reduce/inhibit vasoconstriction strengthens the rationale for iron supplementation interventions to be considered in PAH independently of the patient’s hemoglobin. Of note, the regulatory effects of iron on ET-1 were recapitulated in cultured PASMCs treated with iron, HAMP peptide (to block iron export), or hamp siRNA (to increase iron export), confirming that ET-1 expression in PASMCs responds directly to intracellular iron levels. A search for iron response elements (IREs) in the 3′ UTR and 5′ UTR regions of the et-1 transcript using the SIRES (searching for iron response elements) platform did not yield any results, suggesting that ET-1 response to iron levels may not be IRP-mediated. ET-1 is well-recognized as a HIF-regulated gene and has been shown to be elevated downstream of HIF-2α in mice lacking IRP1, which also develop PAH (34). Intracellular iron deficiency is known to stabilize HIF proteins through limiting the activity of iron- and oxygen-dependent prolyl hydroxylases. While we could not detect up-regulation of other HIF-target genes in iron-deficient PASMCs, this result is entirely consistent with the previous finding that ET-1 was the only HIF-target gene (among 10 targets tested) that increased in the lungs of mice with Chuvash polycythemia which have a moderate normoxic HIF accumulation (35). ET-1 is also a vasoconstrictor in the systemic circulation, and we did explore systemic hemodynamics in fpnC326Y^fl/fl^ SMMHC-CreER^T2+^ mice. However, we found no changes in systemic blood pressure compared with fpnC326Y^fl/fl^ controls (*SI Appendix*, Fig. S6). This is consistent with the observation that the up-regulation of FPN and of ET-1 in fpnC326Y^fl/fl^ SMMHC-CreER^T2+^ mice was seen to be confined to the pulmonary vasculature (*SI Appendix*, Fig. S2*A*). This indicates that HAMP in these cells plays a greater role in the cell-autonomous regulation of FPN than in other SMCs, possibly reflecting site-dependent differences in basal hepcidin expression. Consistent with this, we found basal hamp mRNA expression in primary mouse PASMCs to be higher than in aortic SMCs (*SI Appendix*, Fig. S2*B*).

A third important finding of the current study is that intracellular iron homeostasis in PASMCs requires the action of a cell-autonomous HAMP/FPN axis. Loss of HAMP responsiveness in the PASMCs of fpnC326Y^fl/fl^ SMMHC-CreER^T2+^ mice resulted in increased FPN expression, intracellular iron deficiency, and remodelling of the pulmonary arteries, giving rise to PAH. The posttranslational regulation of FPN by HAMP in PASMCs appears to be a nonredundant component of cellular iron homeostasis, because up-regulation of tfr1 and dmt1 was not sufficient to maintain normal PASMC function. The regulation of FPN in PASMCs by HAMP has been reported before in an in vitro setting (22), but the current study is an in vivo demonstration that this regulation is important for the normal physiology of the pulmonary vasculature.

HAMP and FPN are best known for regulating systemic iron availability through the control of iron flux from the duodenal enterocytes, splenic macrophages, and hepatocytes, the sites of iron absorption, recycling, and storage, respectively (16–18). This study in PASMCs is only the second example of a physiologically important role for a local HAMP/FPN axis. The first example was in cardiomyocytes, where previous work from this laboratory demonstrated that HAMP-dependent cell-autonomous control of cardiomyocyte iron levels is required for normal cardiac function (23, 24). Together, these studies produce a paradigm shift in our understanding of the functions of the HAMP/FPN axis and further reveal that this pathway is particularly important for normal cardiovascular physiology. Additionally, previous cardiac work from this laboratory revealed that there is an interplay between the local and the systemic HAMP/FPN axes. For instance, it was found that ubiquitous loss of HAMP or HAMP responsiveness compensated, through increased systemic iron availability, for the otherwise detrimental effects of loss of cellular HAMP or HAMP responsiveness in cardiomyocytes (23, 24). Similarly, RVSP and right heart function in mice with a ubiquitous loss of HAMP responsiveness (fpnC326Y-KI) are preserved.

There is an association, the precise nature of which is not fully understood, between perturbations in serum HAMP levels and PAH. On the one hand, elevated serum HAMP and iron deficiency are prevalent in PAH patients, possibly driven by inflammation (hepcidin is up-regulated by interleukin-6) (2–5, 31). On the other hand, PAH is also prevalent in patients with β-thalassaemia, which have inappropriately low HAMP levels (due to suppression by erythroferrone) and consequently iron overload (36, 37). This work supports the notion that PASMC iron levels are determined by a fine balance between systemic iron serum availability (controlled by the systemic HAMP/FPN axis) and cellular iron efflux (controlled by the local HAMP/FPN axis). The effects of inflammation, BMPR2 mutations and erythroferrone on iron levels in PASMCs would therefore be the sum of changes in iron fluxes through both the systemic and the local HAMP/FPN axes (Fig. 5*B*). Here we also provide evidence that BMPR2 mutations are associated with reduced hepcidin expression in PASMCs, and consequently increased FPN and ET-1 levels. These effects could constitute an additional mechanistic link between BMPR2 mutations and familial PAH. Further studies are warranted to build a better understanding of how perturbations in the HAMP/FPN axis contribute to PAH pathophysiology. Currently, there is considerable interest in targeting the HAMP/FPN axis in diseases of systemic iron deficiency and iron overload. Our studies suggest that such strategies may also impinge on PASMC intracellular iron homeostasis and on the function of the pulmonary vasculature.

## Methods

### Mice.

All animal procedures were compliant with the UK Home Office Animals (Scientific Procedures) Act 1986 and approved by the University of Oxford Medical Sciences Division Ethical Review Committee.

The conditional fpnC326Y^fl^ allele was generated as described previously (24). Experimental animals were produced as described in *SI Appendix*. In experiments involving delivery of iron in vivo, mice were injected i.v. with 100 μL saline containing 0.5 mg iron in the form of ferric carboxymaltose (Ferinject, Vifor Pharma). The selective endothelin subtype-A receptor antagonist BQ-123 (product code B150, Sigma) was dissolved in sterile saline at a concentration of 1.89 mmol/L and delivered at a rate of 200 nmol/kg/d using s.c. implanted osmotic minipumps (Alzet).

### Human PASMCs.

The normal human PASMC cell line (PCS-100-023) was purchased from ATCC and cultured in DMEM containing Hanks salts, l-glutamine, antibiotics, and 5% FCS. Details of PASMCs from PAH patients and healthy controls PASMCs are provided in *SI Appendix*.

### Mouse PASMCs.

Adult mouse pulmonary arteries were dissected out in dissociation medium (DM). The composition of the DM is described in *SI Appendix*. Enzymatic digestion was carried out as described in *SI Appendix*. Cells were cultured in DMEM containing Hanks salts, l-glutamine, antibiotics, and 5% FCS.

### In Vitro Treatments.

Mouse PASMCs were treated with mouse HAMP peptide (PLP-4434-s, Peptides International) while human PASMCs were treated with human HAMP peptide (PLP-4392-s, Peptides International) at a final concentration of 0.5 μmol/L for 12–16 h. Ferric citrate (F3388, Sigma-Aldrich) was dissolved in cell growth medium at a final concentration of 200 μmol/L. Deferroxamine (D9533, Sigma-Aldrich) was used at 100 μmol/L. Human and mouse hamp siRNA (Silencer Select assay code S195324 and S96551, respectively, Thermo Fisher Scientific) were transfected into PASMCs at a concentration of 50 nmol/L using Lipofectamine 2000 (11668027, Thermo Fisher Scientific) according to manufacturer’s instructions.

### Gene Expression.

Total RNA extraction and cDNA synthesis were carried out as previously described (23, 24) and detailed in *SI Appendix*.

### Enzyme-Linked Immunoassay.

ET-1 in supernatants and human plasma was measured using Human Endothelin-1 Quantikine ELISA Kit (DET100) according to the manufacturer’s instructions. Human and mouse HAMP in supernatants were measured by HAMP ELISA (DHP250 and LS-F11620, respectively).

### Immunostaining.

Tissues were prepared for histology as described previously (23, 24). Details of antibodies and microscopy are provided in *SI Appendix*.

### RVSP Measurements.

Mice were anesthetized with 2% isofluorane in O_2_ and ventricular systolic pressure was assessed by catheterization using a Millar SPR-869 pressure-volume catheter.

### Cine MRI.

Parameter of cardiac function was measured in anesthetized mice (2% isofluorane in O_2_) as described previously (23, 24).

### Iron Indices.

Serum iron and ferritin levels, hemoglobin, and tissue iron were determined as described previously and outlined in *SI Appendix* (23, 24).

### Statistics.

Values are shown as mean ± SEM. Paired comparisons were performed using Student’s *t* test. Multiple comparisons were drawn using ANOVA. Post hoc tests used Bonferroni correction.

## Supplementary Material

Supplementary File
