# Supplementary material for: Intracellular iron deficiency in pulmonary arterial smooth muscle cells induces pulmonary arterial hypertension in mice
Source: Proc Natl Acad Sci U S A. 2019 May 31;116(26):13122–30. doi: 10.1073/pnas.1822010116 (PMC6600981; doi:10.1073/pnas.1822010116)
Supplement: Supplementary File [file pnas.1822010116.sapp.pdf]

SI Appendix

1. SUPPLEMENTAL FIGURES

Fig S1

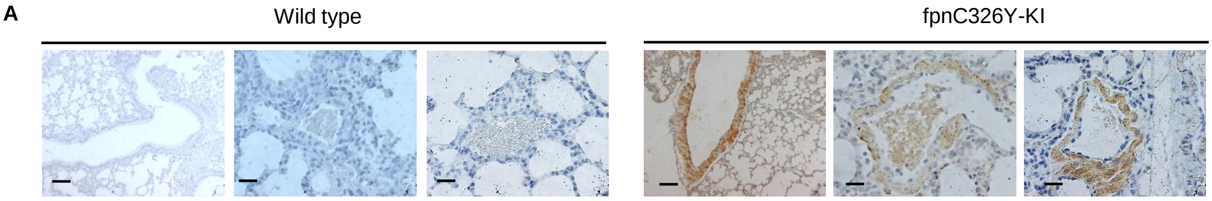

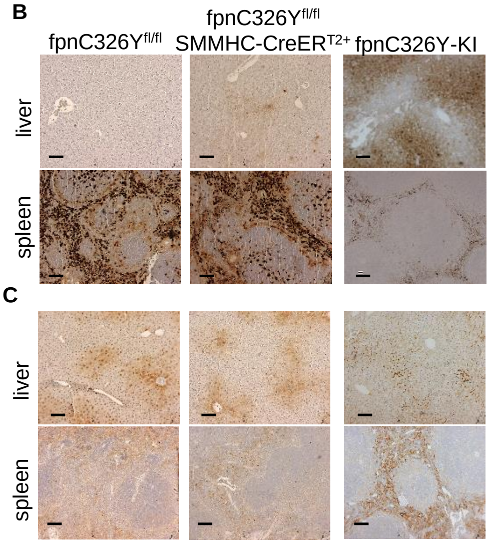

D

	fpnC326Y$^{fl/fl}$	fpnC326Y$^{fl/fl}$ SMMHC-CreER^{T2+}	fpnC326Y-KI
liver iron (ppm)	56.8 ± 4.5	62.8 ± 3.9	295.1 ± 36.4*
spleen iron (ppm)	919.0 ± 213.3	906.3 ± 149.5	527.9 ± 83.9*
lung iron (ppm)	89.9 ± 9.4	95.5 ± 9.9	170.1 ± 25.5*
serum iron (µmol/L)	47.0 ± 7.2	52.6 ± 2.9	76.7 ± 4.7*
ferritin (µg/L)	599.8 ± 73.2	464.1 ± 80.0	958.6 ± 135.8*
hemoglobin (g/L)	137.9 ± 5.8	147.1 ± 4.9	140.6 ± 2.2

Figure S1-Loss of HAMP responsiveness in PASMCs does not affect systemic iron control. **A**. Representative images of FPN immunostaining in 3-month old fpnC326Y-KI mice and wild type littermate controls. Original magnification x10, scale bar 100um (left panel) and x40, scale bar 25um (middle and right panels).N=5 per group. **B**. Representative images of DAB-enhanced Perls iron staining in liver and spleen of 3-month old male fpnC326Y$^{fl/fl}$, fpnC326Y$^{fl/fl}$ SMMHC-CreER^{T2+} and fpnC326Y-KI mice. Original magnification x10, scale bar 100um. **C**. Representative images of FPN immunostaining in liver and spleen of 3-month old male fpnC326Y$^{fl/fl}$, fpnC326Y$^{fl/fl}$ SMMHC-CreER^{T2+} and fpnC326Y-KI mice. Original magnification x10, scale bar 100um. N=5 per group. **D**. Tissue iron content and serum iron indices in 3-month old male fpnC326Y$^{fl/fl}$ (n=5), fpnC326Y$^{fl/fl}$ SMMHC-CreER^{T2+} (n=5) and fpnC326Y-KI (n=5) mice. Values are shown as mean±S.E.M. *p<0.05 relative to fpnC326Y$^{fl/fl}$. p values were calculated using ANOVA. Post-hoc test used Bonferroni correction to allow for multiple comparisons.

Fig S2

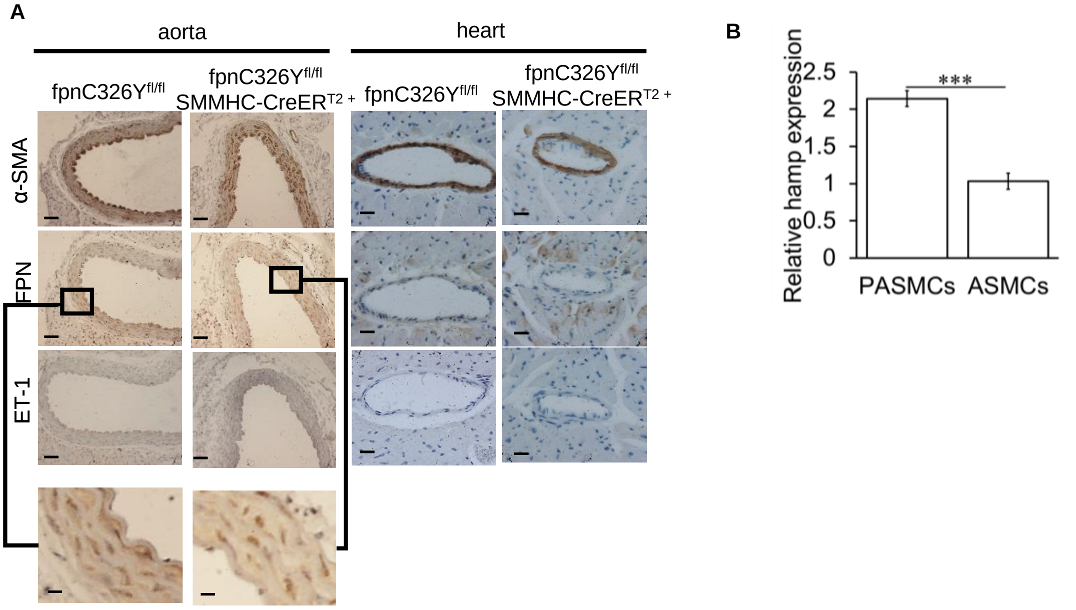

Figure S2- FPN and ET-1 levels are unaltered in systemic smooth muscle cells.
A. Representative images of α-SMA, FPN and ET-1 immunostaining in fpnC326Y$^{fl/fl}$ SMMHC-CreER^{T2+} mice and fpnC326Y$^{fl/fl}$ controls at 12 weeks post-tamoxifen induction (n=6 mice per group). Aorta- Top 3 panels, original magnification x20. Scale bar 50 um. Lower panel, original magnification 100x. Scale bar 10 um. Heart- Original magnification x40. Scale bar 25um. **B.** Relative hamp mRNA expression in freshly-isolated PASMCs and aortic SMCs (ASMCs) isolated from 12 week old C57BL6 mice. N=3 mice, n=3 biological replicates per mouse. Values are shown as mean±S.E.M. p values for paired comparisons were calculated using Student's T test. ***p<0.001.

Table S1- Parameters of LV function

	6 weeks		12 weeks	
	fpn C326Y$^{fl/fl}$	fpn C326Y$^{fl/fl}$ SMMHC-CreER^{T2+}	fpn C326Yf$^{l/fl}$	fpn C326Y$^{fl/fl}$ SMMHC-CreER^{T2+}
**LVEDV (ul)**	58.97 ± 6.35	52.85 ± 3.53	65.29 ± 6.50	61.81 ± 1.63
**LVESV (ul)**	19.55 ± 2.31	19.09 ± 1.36	19.49 ± 3.35	22.01 ± 1.37
**LVEF (%)**	66.46 ± 4.04	63.77 ± 1.29	70.14 ± 4.86	64.38 ± 1.96

Table S1- Parameters of LV function are not different between mice of the two genotypes.
Measurements of left ventricular ejection fraction (LVEF), left ventricular end systolic volume (LVESV) and left ventricular end diastolic volume (LVESD) in fpnC326Y$^{fl/fl}$ SMMHC-CreER^{T2+} mice and fpnC326Y$^{fl/fl}$ controls at 6 weeks and 12 weeks post-tamoxifen induction. N=5-12 per group. Values are shown as mean±S.E.M.

Fig S3

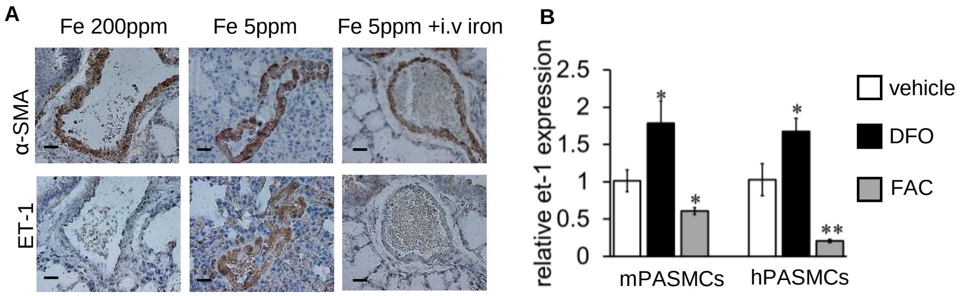

Figure S3- ET-1 is regulated by iron.
A. Representative images of α-SMA and ET-1 immunostaining in C57B6 mice fed a normal chow diet (Fe 200ppm), or an iron-deficient diet (Fe 5ppm) for 6 weeks from weaning. Some of the mice on the iron-deficient diet also received 3 fortnightly i.v injection of ferric cardboxymaltose delivering 0.5mg iron each (Fe 5ppm + i.v iron). n=3 animals per group. Original magnification x40. Scale bar 25um. **B**. Relative et-1 mRNA expression in primary mouse and human PASMCs treated with vehicle, deferroxamine (DFO) or ferric citrate (FAC). Values are shown as mean±S.E.M. p values were calculated using ANOVA. Post-hoc tests used Bonferroni correction to allow for multiple comparisons. *p<0.05. **p<0.01.

Fig S4

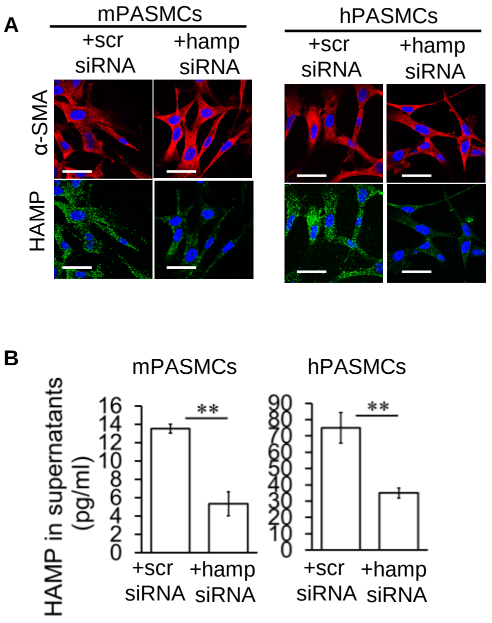

Figure S4- HAMP is expressed in human and mouse PASMCs.
A. Representative images of HAMP staining in mouse and human PASMCs treated with scrambled siRNA (scr siRNA) or hamp siRNA. Original magnification 100x. Scale bar 20 um. **B.** HAMP levels in corresponding supernatants. p values for paired comparisons were calculated using Student's T test. **p<0.01. n=3 biological replicates..

Fig S5

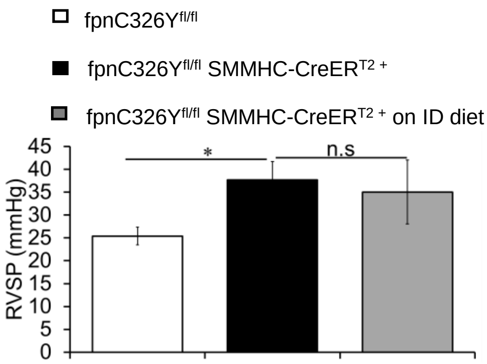

Figure S5- Dietary iron restriction does not induce further increase in RVSP in fpnC326Y$^{fl/fl}$ SMMHC-CreER^{T2+} mice.

Measurements of RVSP at 6 weeks post tamoxifen induction. fpnC326Y$^{fl/fl}$ SMMHC-CreER^{T2+} mice were either fed a control chow diet containing 200ppm iron (n=5), or an iron-deficient (ID) diet containing 5ppm iron (n=5) during the entire 6 weeks. Development of anaemia in animals fed the ID diet was confirmed by haemoglobin measurement. RVSP in control fpnC326Y$^{fl/fl}$ mice at the same time point are also shown. Values are shown as mean±S.E.M. p values were calculated using ANOVA. Post-hoc tests used Bonferroni correction to allow for multiple comparisons*$p<0.05$.

Fig S6

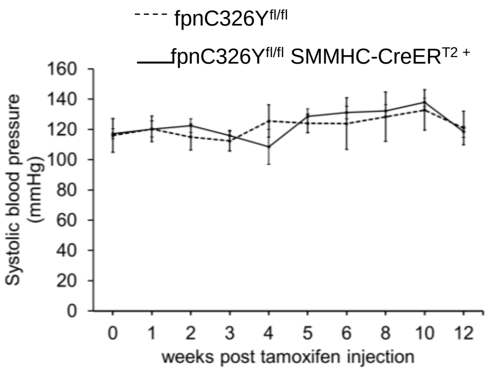

Figure S6- Systemic blood pressure is not altered in fpnC326Y$^{fl/fl}$ SMMHC-CreER^{T2+} mice. Measurements of systolic blood pressure in fpnC326Y$^{fl/fl}$ SMMHC-CreER^{T2+} mice (n=11) and fpnC326Y$^{fl/fl}$ controls (n=6). Measurements were carried out using tail-cuff system in wake mice between 8am and 10am. Mice were trained daily for a week before first measurement. Values are shown as mean±S.E.M.

2. SUPPLEMENTAL METHODS

Mice

Female fpnC326Y^{fl} mice were mated with SMMHC-CreER^{T2} males, which are transgenic for Y chromosome-linked CreER^{T2} gene under control of smooth muscle myosin heavy chain polypeptide 11 (myh11) promoter (1). After 2 rounds of breeding, male fpnC326Y$^{fl/fl}$ SMMHC-CreER^{T2+} mice were identified and mated with female fpnC326Y$^{fl/fl}$ mice to generate fpnC326Y$^{fl/fl}$ SMMHC-CreER^{T2+} males used in experiments. Age-matched fpnC326Y$^{fl/fl}$ control males were generated from breeding fpnC326Y$^{fl/fl}$ males and females. Experimental fpnC326Y$^{fl/fl}$ SMMHC-CreER^{T2+} males and age-matched fpnC326Y$^{fl/fl}$ control males were induced between 3 and 4 weeks of age, using a total of 3 intraperitoneal injections delivering 1mg tamoxifen every second day.Unless otherwise stated, mice were fed a standard chow diet containing 200ppm iron. In systemic iron deficiency studies, animals were fed an iron-deficient diet (5 ppm iron; Teklad TD.99397; Harlan Laboratories), or a matched control diet (200 ppm iron; Teklad TD.08713) from weaning for six weeks.

Acute hypoxia human study

Each study day entailed a 6-hour eucapnic hypoxic exposure (oxygen end-tidal partial pressure 55 mmHg) in a normobaric chamber. On the first study day, immediately before commencement of the hypoxic exposure, 0.9% saline was administered i.v., and on the second, 15 mg/kg (maximum 1 g) ferric carboxymaltose (Ferinject, Vifor Pharma) was added to an appropriate volume of 0.9% saline; each infusion was of 250 ml total volume and given over 15 minutes at a rate of 16.7 ml/min. Venous blood was sampled before each infusion and at 6 hours. Plasma was obtained by centrifugation and immediately frozen at –80°C.

PASMCs with BMPR2 mutations

Cells were a kind gift from Prof N Morrell, University of Cambridge. Mouse PASMCs were obtained from mice with a ubiquitous knock-in of R899X mutation (2). Human PASMCs were harvested from explanted lung tissue obtained from healthy controls and PAH patients with BMPR2 mutations: R899X, W9X and W930S. Patient demographics are detailed in Table S2.

Table S2: Demographics of PAH patients

patient	age	sex	BMPR2 mutation
PAH1	22	M	W9X
PAH2	30	F	R899X
PAH3	39	F	W903S

Dissociation medium for mouse pulmonary arteries

NaCl (110mmol/L), KCl (5mmol/L), Hepes (10mmol/L), KH_2PO_4 (0.5mmol/L), NaH_2PO_4 (0.5mmol/L), $NaHCO_3$ (10mmol/L), taurine (10mmol/L), EDTA (0.5mmol/L), d-glucose (10mmol/L), $CaCl_2$ (0.16 mmol/L), $MgCl_2$ (2 mmol/L), phenol red (0.03 mmol/L); pH adjusted to 7.0 with NaOH.

Isolation of mouse PASMCs

Following isolation in DM, mouse pulmonary arteries were digested by addition of 30 mg/ml papain (product code P4762, Sigma-Aldrich) for 1 h at 4°C, followed by 6 min incubation at 37°C in the presence of 1 mg/ml dithiothreitol (product code D0632, Sigma-Aldrich), then a further incubation with 20 mg/ml Collagenase (C2674, Sigma-Aldrich) for 5 min at 37°C. Tissues were washed in ice cold DM and gently triturated to release cells from the tissue matrix.

Gene expression

Gene expression was measured by quantitative real-time PCR, using Applied Biosystems Taqman gene expression assay probes for dmt1, tfr1 and et-1 and house-keeping gene β-Actin (Life Technologies*, Carlsbad, CA*). The CT value for the gene of interest was first normalised by deducting CT value for β-Actin to obtain a delta CT value. Delta CT values of test samples were further normalised to the average of the delta CT values for control samples to obtain delta delta CT values. Relative gene expression levels were then calculated as $2^{-\text{delta deltaCT}}$.

Antibodies

Sections of formalin-fixed paraffin embedded (FFPE) tissues were stained with the one of the following primary antibodies: rabbit polyclonal anti-mouse FPN antibody (NBP1-21502, Novus biologicals) at 1/200 dilution, rabbit polyclonal anti- mouse alpha smooth muscle Actin antibody (ab5694, Abcam) at 1/500, rabbit polyclonal anti-mouse endothelin1 antibody (ab117757, Abcam) at 1/500 dilution. Secondary antibody staining with HRP-conjugated anti rabbit IgG antibody was carried out using Rabbit EnVision+ System-HRP (K4011, Dako). Sections were visualised using a standard light microscope. For fluorescent staining, PASMCs were cultured on poly-L-lysine pre-coated coverslips, then washed and fixed in 4% PFA for 20min. They were co-stained with rabbit polyclonal anti-mouse FPN antibody (NBP1-21502, Novus biologicals) at 1/200 dilution and Cy3-conjugated anti- mouse alpha smooth muscle Actin antibody (A2547 Sigma-Aldrich) at 1/500. Alexa488-conugated anti-rabbit antibody (ab150073, Abcam) was then used 1/500 as a secondary antibody for the detection of FPN staining. Following counter stain with DAPI, coverslips were visualised using a Fluoview FV1000 confocal microscope (Olympus). For flow cytometry, cells were detached using cell scraper and stained with anti-mouse FPN antibody (NBP1-21502, Novus biologicals) at 1/200 dilution, using Alexa488-conugated anti-rabbit antibody (ab150073, Abcam) at 1/500 as a secondary antibody. Flow cytometry was carried out using the BD Fortessa X20 (BD Biosciences) and the data analysed by FlowJo (LLC).

Quantitation of vessel muscularization

One hundred pulmonary arterioles per section were analysed. Each vessel was assigned either as non-muscularized (no α-SMA staining), partially muscularized (thin or discontinuous layer of α-SMA staining), or fully muscularized (thick continuous layer of α-SMA staining). The percentage distribution of each was calculated per group.

Determination of RV and cardiomyocyte hypertrophy

Hearts were isolated from PBS-perfused fpnC326Y$^{fl/fl}$ SMMHC-CreER^{T2+} males and age-matched fpnC326Y$^{fl/fl}$ control males at 6 and 12 weeks post-tamoxifen injection. Hearts were weighed on a precision balance and weights recorded before and after removal of the RV. Values were expressed as RV/LV+septum. For determination of cardiomyocyte size, FFPE heart sections were stained for wheat germ agglutinin WGA (W32464, Thermosfisher) at 5ug/ml, and counterstained with DAPI. Images of WGA-stained RVs were analysed by Image J to calculate the average size of RV cardiomyocytes for each mouse.

Iron indices

Serum iron and ferritin levels were determined using the ABX-Pentra system (Horiba Medical, CA). Determination of total elemental iron in tissues from PBS-perfused animals was carried out by inductively coupled plasma mass spectrometry (ICP-MS) as described previously (3, 4). Haemoglobin was recorded from fresh blood using the HemoCue Hb 201+ system. Ferritin levels in cell lysates were measured by ELISA (orb408073, Biorbyt for mouse ferritin and LS-F5312, lsbio for human ferritin) according to the manufacturer's instructions.

References

1. Wirth A, Benyo Z, Lukasova M, Leutgeb B, Wettschureck N, Gorbey S, Orsy P, Horvath B, Maser-Gluth C, Greiner E, Lemmer B, Schutz G, Gutkind JS, Offermanns S (2008) G12-g13-larg-mediated signaling in vascular smooth muscle is required for salt-induced hypertension. *Nat Med* 14:64-68
2. Long L, Yang X, Morrell N, Southwood M (2011) S97 BMPR2 R899X knock-in mice developed age-related pulmonary hypertension. *Thorax* 66(Suppl 4):A46-A46
3. Lakhal-Littleton S, Wolna M, Carr CA, Miller JJ, Christian HC, Ball V, Santos A, Diaz R, Biggs D, Stillion R, Holdship P, Larner F, Tyler DJ, Clarke K, Davies B, Robbins PA (2015) Cardiac ferroportin regulates cellular iron homeostasis and is important for cardiac function. *Proc Natl Acad Sci U S A* 112:3164-3169
4. Lakhal-Littleton S, Wolna M, Chung YJ, Christian HC, Heather LC, Brescia M, Ball V, Diaz R, Santos A, Biggs D, Clarke K, Davies B, Robbins PA (2016) An essential cell-autonomous role for hepcidin in cardiac iron homeostasis. *Elife* 5
